# Real-Time Impedance Monitoring During Electroporation Processes in Vegetal Tissue Using a High-Performance Generator

**DOI:** 10.3390/s20113158

**Published:** 2020-06-02

**Authors:** Borja López-Alonso, Héctor Sarnago, Óscar Lucía, Pablo Briz, José Miguel Burdío

**Affiliations:** Department of Electronic Engineering and Communications, University of Zaragoza, 50018 Zaragoza, Spain; hsarnago@unizar.es (H.S.); olucia@unizar.es (Ó.L.); 723669@unizar.es (P.B.); burdio@unizar.es (J.M.B.)

**Keywords:** irreversible electroporation, pulse generators, biologic tissue

## Abstract

Classical application of electroporation is carried out by using fixed protocols that do not clearly assure the complete ablation of the desired tissue. Nowadays, new methods that pursue the control of the treatment by studying the change in impedance during the applied pulses as a function of the electric field are being developed. These types of control seek to carry out the treatment in the fastest way, decreasing undesired effects and treatment time while ensuring the proper tumour ablation. The objective of this research is to determine the state of the treatment by continuously monitoring the impedance by using a novel versatile high-voltage generator and sensor system. To study the impedance dynamics in real time, the use of pulses of reduced voltage, below the threshold of reversible electroporation, is tested to characterise the state-of-the-treatment without interfering with it. With this purpose, a generator that provides both low voltage for sense tissue changes and high voltage for irreversible electroporation (IRE) was developed. In conclusion, the characterisation of the effects of electroporation in vegetal tissue, combined with the real-time monitoring of the state-of-the-treatment, will enable the provision of safer and more effective treatments.

## 1. Introduction

Electroporation is a phenomenon based on the change in the permeability of the cell membrane by applying an electric field. There are mainly two types: the reversible electroporation, which is commonly used as a physical drug delivery system [1], and the irreversible electroporation (IRE), where the applied field is enough to cause irreversible tissue damage [2].

Although IRE is currently being used in many contexts, including industrial applications for food processing and/or sterilization, this paper will be focused on IRE as one of the most promising therapies for cancer treatment [3,4]. In the past, several methods have been reported for calculating the treated volume in a given treatment [5] as well as the electrical protocols for the treatment application [6,7]. This requires, however, 3D finite element simulation [8,9,10] to estimate the treated area, and no real-time monitoring is performed. As a consequence, this requires oversizing the injury created to avoid untreated areas. After the treatment planning, a fixed number of pulses is usually applied. If the treatment is considered to have been ineffective, it is usually reapplied again entirely.

Nowadays, there are a few commercially available generators for clinical applications of electroporation: Cliniporator^®^ (Capri, Italy) from IGEA and Nanoknife^®^ (New York, NY, USA) from AngioDynamics. In addition to these, there are also additional devices intended for in vitro electroporation, such as the NEPA^®^ (Chiba, Japan) Porator from NEPAGENE, among others. However, these devices are designed for specific treatment techniques and have limited applicability to research purposes with limited output/voltage ratings and limited monitoring capabilities. Their main drawbacks are the limited output voltage and current, which do not allow working with large tissue volumes, and the low ability to control the shape of the output voltage [11]. In this sense, several experimental generators have been developed in the past [12,13,14]. In this paper, a highly versatile generator was designed and used to achieve faster experimentation. It includes an embedded small-signal conductivity measurement device without the need to use additional equipment such as impedance analysers [15,16].The objective of the developed generator is to perform a real-time monitoring of the treatment using the custom designed high performance equipment.

The development of real-time monitoring allows detecting problems during the application of the treatment and controlling the electroporation status more precisely [17,18]. For the development of these tools, it is necessary to know the methods to evaluate the real effect of electroporation.

IRE usually requires extensive experimentation in order to optimize the process and maximize the desired cell destruction with minimum side effects. Besides, unlike purely thermal treatments like radiofrequency or microwave ablation, it requires in vivo experiments to assess its biological effects [19]. For this reason, biological tissues are commonly selected, and vegetable tissues such as potatoes are present in the literature [20,21].

Currently, there are several methods to evaluate the state of the tissue after electroporation. The most commonly used methods in the study of electroporation are histopathology analysis [22,23], and the observation of the colour change produced by natural degradation and the dyeing of the tissue. The histopathological analysis consists of a study of the state of the cells at the microscopic level. On the other hand, dyeing and observation of tissue degradation are techniques that evaluate the treatment status produced by macroscopic visual inspection. They also allow to one to determine treated areas, but not to know the state of the cells. Moreover, histopathology analysis allows one to gain an in-depth knowledge of the cellular state, but it requires expensive and very long processes. Finally, in the field of food processing, other techniques have been widely used to optimize the extraction of certain substances, some of which can be applied to this purpose [24].

A method commonly used to determine the state of the electroporation process in a macroscopic way is based on studying the release of certain ions after electroporation [25]. With this method, it is possible to carry out an extensive experimentation that allows one to study electroporation status by real-time small-signal [26] conductivity monitoring. 

**Figure 1:**
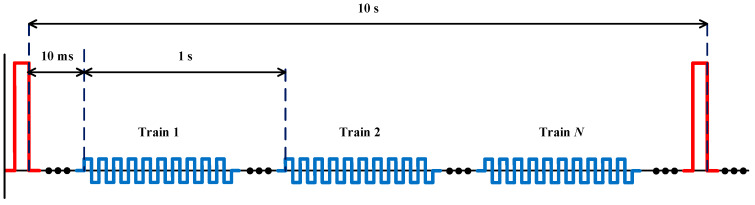
Voltage waveforms used in the experiments including large- and small-signal waveforms.

In this paper, a continuous evaluation of small-signal conductivity monitoring as a method of estimating the state of the conductivity in small-signal in order to determine the state of the electroporation is presented (Figure 1). An experimental method is proposed to estimate tissue status in order to evaluate this technique and a high-performance electroporation generator is developed for this purpose.

The remainder of this paper is organized as follows: Section 2 describes the methods and material that were used in this paper. Section 3 details the different results obtained from the analysis of the different electrical and physiological data extracted from the experiment performed. Finally, Section 4 presents the discussion.

## 2. Materials and Methods

In order to develop an optimized electroporation control strategy, it is necessary to know the different factors related to its application. It also required the use of specific tools depending on the field of study. In this section, the different methods and the tools used in this research are introduced.

### 2.1. High-Voltage Generator

In order to implement the electroporation real-time impedance monitoring system, a new high-voltage generator architecture is proposed. The developed generator is a multilevel converter composed of half-bridge power electronic modules based on insulated gate bipolar transistor (IGBT). The developed generator has a 4.5 mF bus capacitor. The minimum impedance measured during this experiment was 18 Ω. This means that, for the worst-case condition, eight simultaneous experiments can be performed at the same time applying 1-ms pulses with a 10% output voltage ripple.

**Figure 2:**
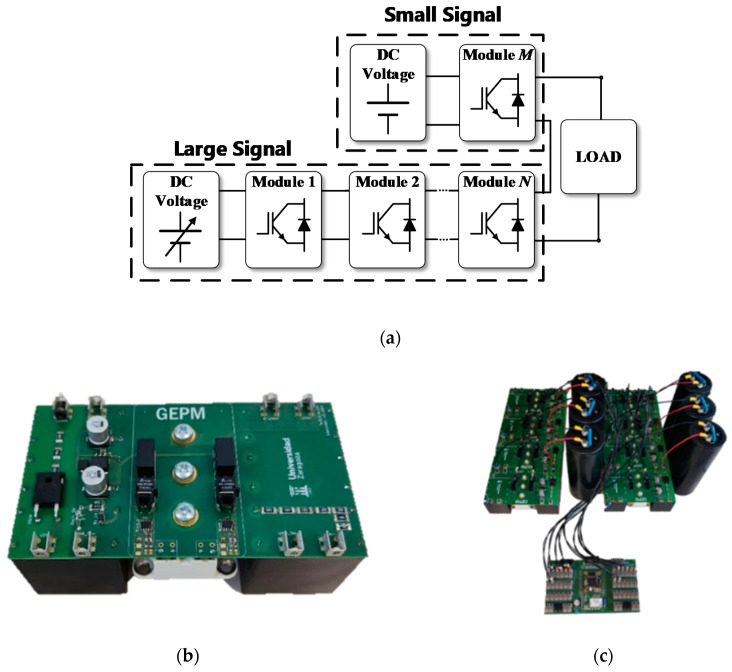
High-voltage generator for electroporation: (**a**) general block diagram, (**b**) single module and (**c**) high-voltage generator used in the experimental measurements.

Figure 2a shows a block diagram of the generator and Figure 2b shows a single-level unit of the experimental prototype. All modules are connected to a single DC power source.

The variable output voltage of the generator will be the sum of the accumulated voltage of the connected modules, in other words, *n*-times the voltage of the connected continuous DC supply. The high-voltage generator presented is robust, simple and versatile. It allows delivering up to 4 kV bipolar output voltage and 500 A with stable output. 

**Figure 3:**
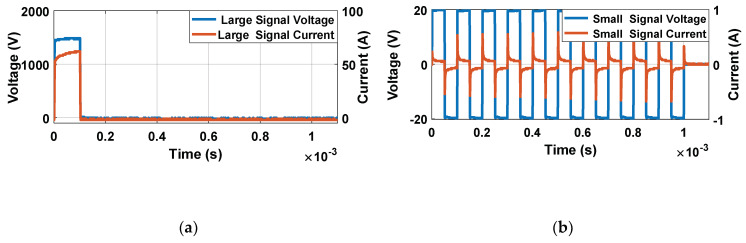
Large- and small-signal voltage (blue) and current (red) pulses: (**a**) large-signal pulses and (**b**) small-signal pulses.

First, in order to ensure the proper irreversible electroporation treatment, a high-voltage pulse can be delivered by the generator, as shown in Figure 3a. Secondly, in order to control the treatment, a set of low-amplitude pulses is applied to perform the real-time impedance monitorization (Figure 3b).

The experimental test-bench is implemented taking into account the considered requirements. In order to avoid any effect caused by a component mismatch and to ensure a reliable operation, the same power blocks (Figure 2a) are considered for both high amplitude and low amplitude pulses.

Figure 2c shows the final high-voltage generator test-bench, which includes bulky electrolytic capacitors to ensure a constant voltage during the treatment independently of the load impedance.

### 2.2. Data Processing

As previously mentioned, one of the objectives of this research is the real-time monitoring of the treatment. Currently, it is difficult to quantify the treatment performed to each one of the cells in an accurate way and, consequently, it is even more challenging doing it in real time. For these reasons, one of the aims of this research is to propose methods that allow a real-time control of the treatment in a macroscopic way. The simplest and most extended way is to study the treatment status based on the change in tissue conductivity. This change is usually measured by the voltage and current at the end of each electroporation pulse. 

This type of measurement depends on the pulsation period and does not allow continuous monitoring of the treatment. It only allows the monitoring of a single impedance value and does not analyse its frequency behaviour. Besides, continuous measurement allows a more effective control of the treatment, and also detects problems during the application. This allows the application of a safer and more effective treatment. Moreover, the data regarding the impedance change as a function of frequency provides more information about the state of the electroporation process. 

Considering the versatile generator designed, the measurement pulses are shown in Figure 3b, composed of a square wave train with 10 bipolar pulses. The objective of using a bipolar waveform to carry out the impedance measurements is to eliminate the DC value of the signal, minimizing its undesired effects in the tissue. The amplitude of these characterisation pulses is always below the electroporation threshold, and they communicate the minimum possible energy to reduce the delivering effect.

While there are several methods to process the small-signal pulses, in this case, the Fourier transform was selected. The expression of the harmonic content of the applied voltage is as follows:

(1) $$\begin{array}{l} f(t)=\sum_{n=1}^{\infty }\frac{4V}{n\pi }\sin(n2\pi f_{0}t),\\ n=(1,3,5,7,9\ldots odd). \end{array}$$

**Figure 4:**
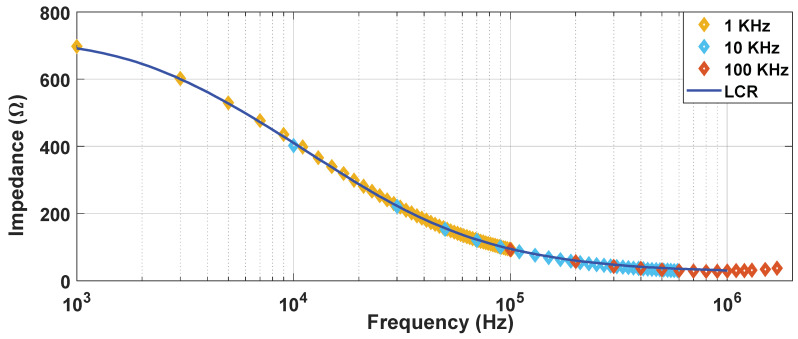
Module of the impedance measurement: comparison of the measurements obtained by processing the data obtained from the proposed generator at different frequencies and by a precision impedance analyser (LCR).

This equation represents the Fourier series decomposition of a zero-centred square wave with peak voltage *V.* The spectrum of this wave is composed of *n* harmonics, whose frequencies are equal to *n* times the fundamental frequency of the square wave, *n* being odd. Figure 4 shows the impedance spectrum reconstructed using these measuring pulses at different frequencies. It was determined that a square wave of 10 kHz enables continuous monitoring of the treatment in the desired frequency spectrum. By doing so, it is possible to reconstruct the conductivity spectrum range (10 kHz to 1 MHz) without the need of external equipment such as an impedance analyser (LCR) which may require off-line measurements.

### 2.3. Experimental Evaluation

One of the key aspects of this research is to find a direct correlation between the electroporation treatment evolution and the variation of electrical parameters. For this purpose, this research takes as a starting point some techniques previously applied in the field of food processing. These techniques allow a large number of experiments to be carried out in a simple way, and also enable observing the effect of the treatment in a comparative way. Therefore, if the treated volume is adequately controlled and adequate tissue characterisation is performed, they can be used to correlate the extracted electrical data.

In this paper, an experimental method was developed to evaluate the change caused in the tissue status by means of electroporation. This method is based on the analysis of the release of different ions when the cells are electroporated.

Depending on the extracted substances, the status of the tissue can be estimated. To measure the various substances that were extracted, different physical and chemical methods can be used, but these are normally based on the detection of a single substance and can be expensive and complex. For these reasons, the measurement of the electrical conductivity of the medium in which the substances are immersed is proposed.

The setup used during the experiment is composed of three parts:

- High-voltage generator. This test-bench subset consists of the high-voltage generator previously described, an 8-bit LeCroy oscilloscope Wavesurfer 3024, two differential voltage probes LeCroy HVD3206 and two model 110 Pearson^tm^ current monitors.
- Precision off-line impedance measurement system. Keysight E4990A (California, United States) impedance analysers with a bandwidth between 20 Hz and 300 kHz are used.
- Experimentation area, where the potato specimens are carved and placed in distilled water.

The different steps that are followed in the proposed experiment:

- Firstly, the selected vegetal tissue is the Monalisa variety potato. Cylindrical potato specimens are cut of 1 cm thick and 3 cm in diameter in order to achieve an adequate electric field distribution and, therefore, a uniform treatment. The electroporation is applied with the electrodes in contact with the potato specimens, that is, with a separation equal to its thickness of 1 cm. With these potato specimens, the applied electric field (V/cm) will be equal to the voltage between the electrodes. The medium used is distilled water with a conductivity of approximately 0.00001 S/m, and the electrodes are composed of gold-plated copper.
- Secondly, the electroporation treatment is applied to potatoes by means of parallel-plate electrodes to achieve a distribution of the electric field as homogeneous as possible. These plates along with the potato are immersed in distilled water. This water is the medium in which the different substances will be released and allows an adequate characterisation of the change in electrical conductivity. The water also allows us to control the temperature and reduces the thermal effects. To achieve an additional decrease in the thermal effects, the applied high-voltage pulses will have a spacing of 10 s, as represented in Figure 1.
- After application of the treatment, the potato specimens are released from the electrodes and placed in the sample cup to expose the largest possible surface area to distilled water. This allows the release of substances to occur as quickly and homogeneously as possible.
- It is established that the potatoes must remain in distilled water for an hour and a half before taking them out.
- Finally, the conductivity measurement in the medium is carried out by means of a precision impedance analyser.

## 3. Results

### 3.1. Electrical Data Results

**Figure 5:**
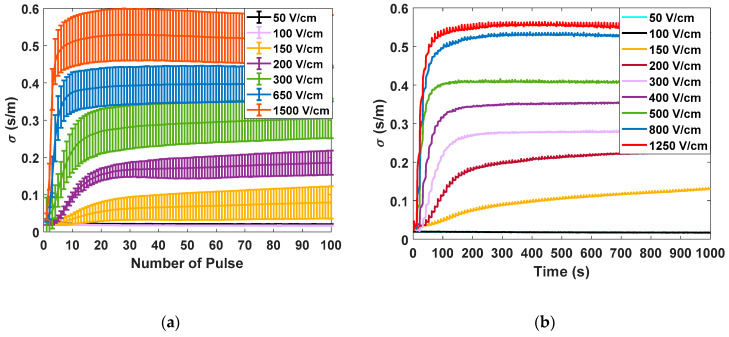

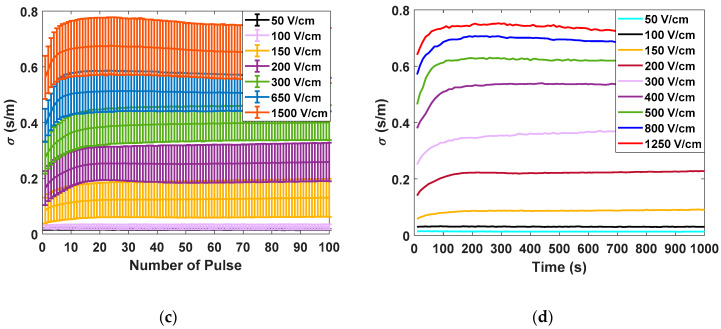
Large-signal and small-signal conductivity measurements for different electric field intensities: (**a**) average and typical deviation of small-signal conductivity measured 10 ms after the application of each electroporation pulse, taking ten samples per studied electric field; (**b**) small-signal conductivity measurements for nine samples between each large-signal pulse and nine different electric fields; (**c**) average and typical deviation of large-signal conductivity measured in electroporation pulse for ten samples per studied electric field; and (**d**) large-signal conductivity measurements for nine different electric fields.

Figure 5 shows the different measurements carried out in potato tissue, comparing the data obtained in large- and small-signal measurements using the developed generator. In Figure 5a, the data corresponding to the measurements made with the small-signal pulses are collected. The data corresponding to the application of seven different electric field intensities measured 10 times are represented. All represented small-signal conductivities are evaluated at 10 kHz. In Figure 5b, the complete evolution of the small-signal conductivity is represented by applying the pulse trains, as shown in Figure 5. The objective is to evaluate the complete evolution of the conductivity during the treatment application in order to analyse its dynamics. In Figure 5c, the data corresponding to the mean and standard deviation of the large-signal conductivity measured in each of the electroporation pulses is shown. Finally, in Figure 5d, the complete evolution of the large-signal conductivity is represented for a single case of each applied electric field. 

The following conclusions are extracted from these experimental data. First, for both small-signal and large-signal measurements, a significant standard deviation is observed in the measurements. This variability is a consequence of the complexity of the biologic tissue, both in structure and its difference in composition between different samples. This variability can harden treatment control. The tendency when the treatment is complete is the same in both cases. It is important to note that temperature is a factor that could influence measurements. For this reason, the temperature increase was measured to be limited to 5 °C. Only in experiments in which electric fields greater than 1000 V/cm were applied was a temperature increase of 15 °C observed in the worst case.

The initial dynamics show one difference between the small-signal and large-signal measurements. In the case of the small-signal, the dynamics of the conductivity between the pulses can also be observed due to its continuous online measurement which provides more information.

**Figure 6:**
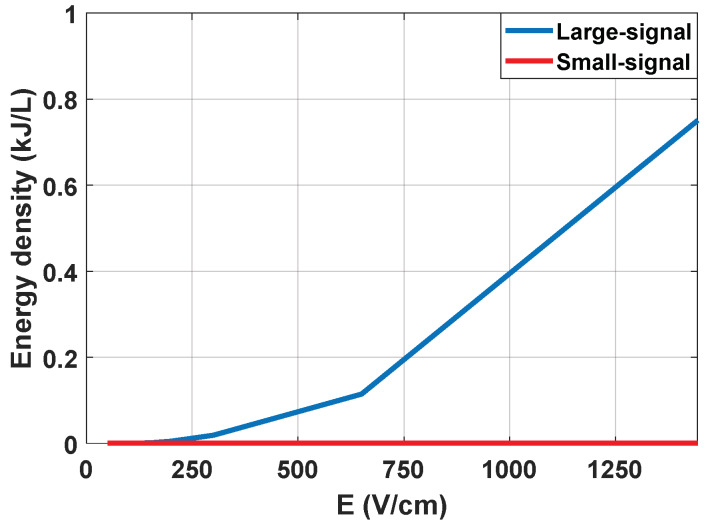
Average energy density applied as a function of the electric field.

It is concluded that the measurement of the small-signal impedance has significant advantages and can be carried out properly by means of the designed equipment. First, it is possible to extract a complete part of the impedance spectrum from both its real and imaginary part. Additionally, it is also a measurement that can be carried out by applying an electric field well below the electroporation limit, and with negligible energy from a thermal point of view, as can be seen in Figure 6.

### 3.2. Tissue Characterisation Results

**Figure 7:**
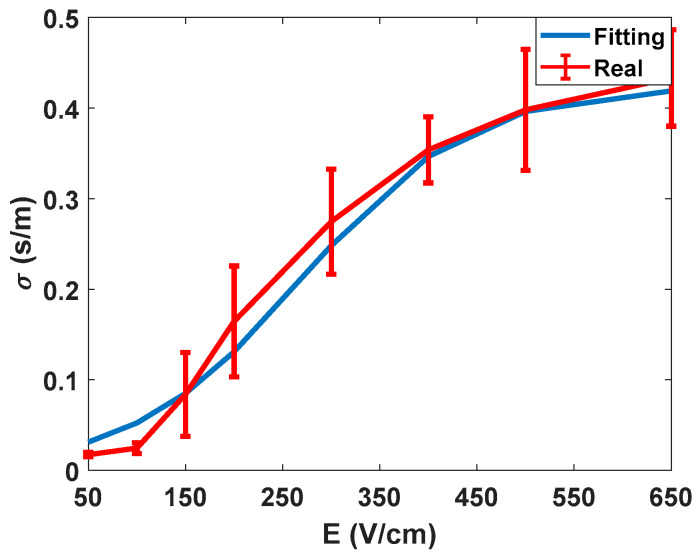
Electrical conductivity of potato tissue as a function of the applied electric field at 10 kHz.

After summarizing the electrical data obtained from experimentation, the data obtained from the characterisation and the proposed method to evaluate the electroporation process is discussed. In Figure 7, the electric characterisation of the potato tissue is represented. As already described in the literature [27], the electrical conductivity of the tissue describes a sigmoid depending on the amplitude of the applied electric field.

Thus, it is possible to establish thresholds at higher and lower electric field ranges. In this specific test, the characterisation was carried out for the conductivity observed at 10 kHz, i.e., the values of the large-signal pulses of 100 µs. These measurements were made to validate the accuracy of the signal processing methodology used, as well as to obtain an initial assessment of the behaviour of the tissue in test conditions.

The next step is to present the data extracted from the experimentation according to the proposed method. This status is estimated by the change in the conductivity of distilled water as a consequence of the release of ions from the treated potato sample.

**Figure 8:**
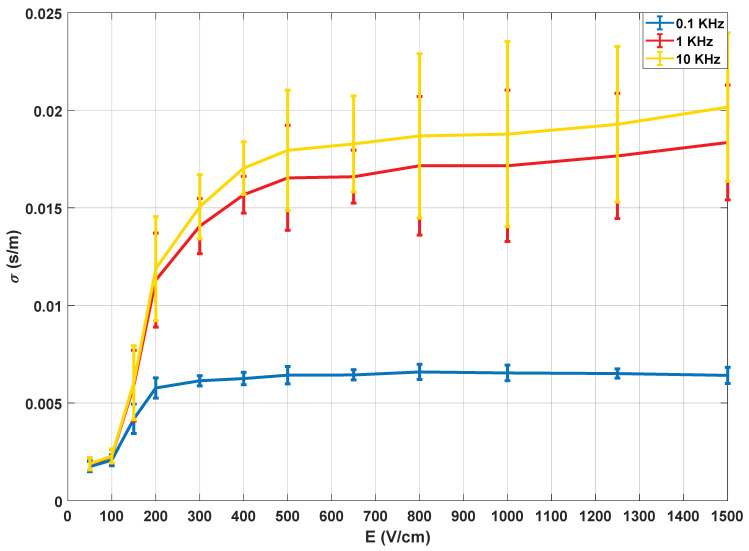
Electrical conductivity of distilled water and potato sample 1.5 h after the electroporation process.

Figure 8 shows the data obtained from the conductivity measured in the water after a residence time of the tissue of 1.5 h.

The collected data comprises impedance measurements between 20 Hz and 200 kHz, with 10 points per decade, and Figure 8 shows the evolution of the conductivity for three different frequencies that are considered representative of the full spectrum.

Just as in the previous case, ten experiments were carried out for each applied electric field, applying 100 pulses of 100 µs with a 10 s pulsation period, in addition to the small-signal characterisation pulses. For the representation, similarly to the previous measurements, the average and the standard deviation of all the values were obtained. 

The conclusion from Figure 8 is that the behaviour of the conductivity of the water with respect to the electric field is similar for the entire frequency spectrum, but at lower frequencies, the dispersion of the measurements appears to be lower. Furthermore, the observed conductivity evolution is similar to that obtained from the previous analysis: there is an initial zone where the tissue is not affected, an intermediate part where the reversible electroporation takes place and, finally, a saturation zone which is attributed to a state of maximum affectation and, therefore, irreversible electroporation. 

These results, as well as those obtained in the analysis of the small-signal electrical conductivity, allow us to establish a clear limit to determine the correct electroporation application and will serve to establish control thresholds in the application of irreversible electroporation, improving current fixed protocols.

### 3.3. Experimental Results Correlation

After analysing the electrical data and the electroporation process evolution, it is necessary to compare the results in order to extract the final conclusions of the experimentation. As previously mentioned in the experimental approach, a total of 12 different electric field intensities applied between 50 V/cm and 1500 V/cm were studied, with ten tests being carried out for each electric field. In each experiment, the small- and large-signal electrical measurements were performed and, after applying the treatment, the samples were processed by means of the proposed procedure to study the change produced in the tissue. 

The purpose of the different electric fields applied is to study the effectiveness of the proposed method in the entire electroporation range and, also, to ensure that the small-signal conductivity measurement system does not interfere with the electroporation process.

**Figure 9:**
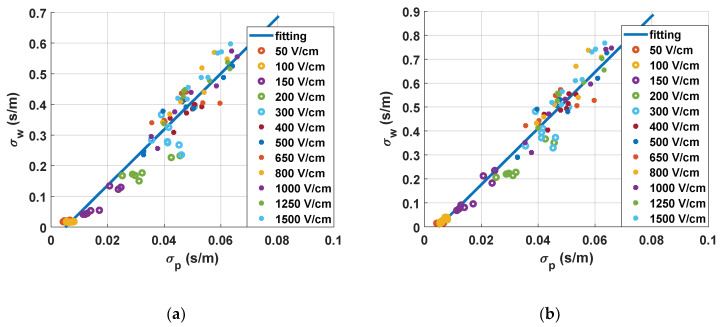
Correlation between the electrical conductivity measured in real time and the electrical conductivity measured in the distilled water after 1.5 h: (**a**) correlation of small-signal conductivity and (**b**) correlation of large-signal conductivity.

In Figure 9, the correlation between the conductivities measured during the treatment and the results obtained from the water conductivity analysis are plotted. 

In Figure 9a, the correlation between the small-signal measurements and the estimation of tissue status is represented. This was extracted by studying the small-signal conductivity measured 10 ms after the last pulse of the treatment. In this chart, a linear fitting is also included. From these fitting results, it becomes clear that it is possible to control the treatment by means of the continuous monitoring of small-signal conductivity. It is also observed that the measurements down to 300 V/cm are well grouped and differentiated, whereas those at higher electric fields are harder to differentiate. This point is where both the saturation of conductivity and the saturation of the electroporation treatment are seen. This enables us to establish this point as an electroporation threshold with the proposed protocol. It can also be observed that there is an adequate correlation between the measurement of small-signal conductivity and the real changes measured in the tissue.

In Figure 9b, the correlation between the large-signal measurements and the estimation of tissue state is represented. This was extracted by studying the large-signal conductivity measured in the last electroporation pulse of the treatment. The statistical tendency of the graph and the conclusions drawn from this observation are consistent. Although both procedures allow us to extract similar information, small-signal conductivity measurements present more advantages in terms of information that can be extracted from the signal and measuring continuity.

## 4. Discussion

Nowadays, electroporation is carried out by preplanning treatments [8,28], and treatment monitoring is carried out by controlling the impedance in large-signal measurements. These tools do not allow real-time monitoring of a treatment, unless the electroporation pulses are applied in a very continuous way. This type of application can only be carried out in very specific situations, since applying electroporation pulses in a continuous way transfers too much energy and causes undesired thermal effects [29,30]. In addition, the treatment of large volumes of tissue is also limited due to the need for applying high-voltage pulses, which are not possible with current state-of-the-art technology [22]. In this paper, it was observed that the proposed monitoring method allows for both long-time monitoring and a large volume of treatments to be carried out, without causing additional damage to the biological tissues, nor interfering in the development of the treatment.

There are mainly two commercially available devices/generators for clinical applications: Cliniporator^®^ from IGEA and Nanoknife^®^ from AngioDynamics [31]. Unlike commercial generators, a high-performance generator [12,14] which is capable of both applying high-voltage electroporation pulses and real-time monitoring of the electroporation process through the application of small-signal pulses was designed (Figure 3). The developed generator, unlike the commercial devices, allows one to carry out the treatment in a very flexible way, making it very suitable for the development of experimentation [11]. The objective is to achieve large ablation as homogeneous as possible and to reduce the time necessary to carry out the experimentation. The generator designed, which is presented in Figure 2, has the total capacity of 11 mF. With this capacity, when looking for a pulse with a maximum variation of 10% of the initial pulse voltage and a duration of 1 ms, it will be possible to connect a load of a minimum of 2.5 Ω. Despite being a reduced version, it is capable of applying pulses of between 1 µs and 1 ms up to a maximum of 1500 V and 500 A. The pulses can be bipolar and can be applied to trains with indefinite separation. The second most interesting section of the developed generator when compared to the current ones is the inclusion of real-time small-signal impedance measurements. This was achieved by using a modular development that allows the parallelization of two independent pulse generator systems, one with a large signal and the other with a small signal, controlled by a joint control module that synchronizes both systems. By using this structure, unlike the generators present in the bibliography [11], a system that carries out both actions simultaneously without the need for external devices was achieved.

In Figure 4, the reconstruction of the impedance spectrum measured by means of small-signal pulses is represented. Unlike the measurements carried out using large-signal pulses, by means of the proposed procedure, it is possible to extract a partial reconstruction of the impedance spectrum without the need for additional equipment [16,17,32].This information can be used to perform a faster and more complex control of the treatment than that which could be achieved by studying the large-signal impedance. As seen in the Figure 5, even using only a single frequency value to compare the small-signal impedance provides much more information [33] than the large-signal impedance, making it more appropriate when it comes to performing a control on the treatment. In view of the results, small-signal impedance monitoring is considered a very interrelated tool to carry out electroporation treatments in a safe and efficient way.

In Figure 9, the results of the experimental methodology based on measuring conductivity changes in the electroporation media are represented. The technique used was based on the research carried out in the field of food engineering. This method allows evaluating the damage on a biological tissue in a comparative and simple way. As can be seen in Figure 9, the change in the conductivity of the fluid is highly dependent on the electric field applied to the tissue, and therefore, it is possible to estimate the state of the tissue. It is important to note that the reversible and irreversible electroporation zones are separated in the graph and are easy to differentiate. To differentiate these areas, we can take into account the tissue characterisation carried out, as well as the behaviours previously described in the bibliography [20]. The dispersion that is seen in the data is considered corrected and due to the complexity of the biological tissues. It is important to note that, in this paper, vegetal tissue was used and, therefore, it is composed of eukaryotic cells with a cell wall and without vacuoles. This tissue is relevant for the application and it has been extensively used in previous research. Moreover, in this paper, it has served to prove the feasibility of the proposed electroporation and monitoring system. However, in order to approximate the experimental results to the targeted treatment, additional in vivo experimentation with animal tissue will be required.

The results of the implementation and experimental analysis show that it is possible to carry out a real-time small-signal [26] conductivity monitoring method to control irreversible electroporation treatment. The proposed electroporation and monitoring system will enable the development of future smart control electroporation systems that enable adaptive delivery and monitoring of electroporation processes, both simultaneously and autonomously [32].

Finally, in this paper, a real-time small-signal conductivity monitoring method to control irreversible electroporation treatments is proposed. It was implemented and experimentally verified. A high-performance generator was designed which is capable of both applying high-voltage electroporation pulses as well as real-time monitoring of the electroporation process through the application of small-signal pulses. An experimental methodology based on measuring conductivity changes in the electroporation media was proposed and experimentally verified to evaluate the effect of the electroporation process in the biological tissue. Lastly, a methodology for online conductivity measurement and processing by a small-signal that allows real-time monitoring of the treatment was proposed. Small-signal and large-signal conductivity measurements were compared and evaluated for continuous monitoring of treatment. These measurements will establish the foundations for improved control strategies for electroporation treatments.
